# Harmonization of Determination of SARS-CoV-2 Antibodies: Is It Always Possible?

**DOI:** 10.3390/diagnostics12020483

**Published:** 2022-02-14

**Authors:** Ruggero Dittadi

**Affiliations:** Laboratory Medicine Unit, Ospedale dell’Angelo, and Regional Center for Biomarkers, Department of Clinical Pathology, Azienda ULSS 3 Serenissima, Mestre, 30122 Venice, Italy; ruggero.dittadi@aulss3.veneto.it

**Keywords:** SARS-CoV-2, antibodies assays, harmonization, COVID-19

## Abstract

A WHO standard was prepared with the aim of harmonizing assays detecting antibodies against SARS-CoV-2, but the issue is currently being debated. We re-evaluated a previously studied set of cases (108 specimens of 48 patients and 60 specimens of 20 vaccinated subjects, collected after 14 days from the first dose and 14 days and 3 months after a second dose of the Comirnaty BNT162b2 vaccine), calculating the ratios between the results of two methods (SARS-CoV-2 IgG anti-RBD, SNIBE, and anti-SARS-CoV-2 QuantiVac ELISA IgG, Euroimmun). In the vaccinated subjects, the ratios of the results between methods according to the WHO standard were relatively dispersed, but the harmonization results were good. On the other hand, in patient samples, the variability between tests was very high, and the harmonization was unsatisfactory (median ratios between methods 2.23, 10th–90th percentile: 1.1–5.6). Interestingly, in patient samples, the harmonization depends on the time from the onset of symptoms and greatly improves after 6 months since the diagnosis. Forty patient specimens and thirty-one of the vaccinated subjects after the second dose were also evaluated with a third method (Access SARS-CoV-2 IgG (1st IS), Beckman Coulter), obtaining a similar trend. We can conclude that the actual effectiveness of harmonization between methods may vary depending on the scenario in which they will be used.

## 1. Introduction

At the end of 2020, the National Institute for Biological Standards and Control (NIBSC) established, using a pooled plasma obtained from individuals who recovered from COVID-19, the first WHO international standard for the immunoglobulins anti-SARS-CoV-2 (NIBSC 20/136). This standard can be used to calibrate in Binding Antibodies Units (BAU) the systems detecting antibodies against SARS-CoV-2 in order to harmonize the different methods [1]. 

Few studies so far have evaluated the real effectiveness of this standard.

Perkmann et al. [2] did not find significant harmonization in samples of vaccinated subjects after the first dose of BNT162b2, measured by five different methods. Lukaszuk et al. [3] only marginally improved the comparison between two methods in vaccinated patients. Infantino et al. [4] evaluated different methods in a mix of patients and vaccinated subjects. They found on average a better comparability between the results but stated that the assays were not interchangeable. 

On the basis of the WHO standard, Ferrari et al. [5] prepared another standard using only vaccinated subjects, with the aim of improving harmonization in this cohort. The results were encouraging, but although the correlations between methods were statistically significant, the distribution of cases remains rather dispersed.

A reason for the difficulty in harmonizing the methods lies in the fact that the different methods are often built to detect antibodies directed towards different epitopes. However, the heterogeneity of antibodies produced at the onset of the disease may be a further cause of misalignment between different methods.

## 2. Methods and Results

In a recently published study [6], we evaluated 108 specimens of 48 patients and 60 specimens of 20 vaccinated subjects, collected after 14 days from the first dose and 14 days and 3 months after a second dose of the Comirnaty BNT162b2 vaccine. We used a method based on the determination of antibodies against receptor-binding domain (SARS-CoV-2 IgG anti-RBD, SNIBE, Shenzen, China) performed on the Maglumi platform and an ELISA method detecting antibodies against protein S1 (anti-SARS-CoV-2 QuantiVac ELISA IgG, Euroimmun, Lubeck, Germany).

We found that the transformation into Binding Antibodies Units allowed us to harmonize the methods only in vaccinated subjects but not in the specimens of patients.

Our original data were re-evaluated by calculating the ratios between the results of the two methods. Using the results expressed in the internal units of each method, the medians of the ratios between ELISA and Maglumi methods were 3.01 in patients, 1.89 in the vaccinated subjects after the first dose, and 1.3 and 1.47, respectively, in vaccinated subjects 14 and 90 days after the second dose.

After the transformation into BAU/mL, the median of the ratios between methods was 2.23 (10th–90th percentile: 1.1–5.6) in patients. In the vaccinated subjects, the ratios after the first dose (median 1.4; 10th–90th percentile 0.93–2.1) were significantly higher (Kruskal–Wallis test: *p* = 0.0009) than in the specimens 14 and 90 days after the second dose (overall median 0.98; 10th–90th percentile 0.78–1.39). 

The comparability between the two methods in vaccinated subjects after the second dose is good and the scattering is reasonably reduced. Less satisfactory was the comparability after the first dose. 

**Figure 1:**
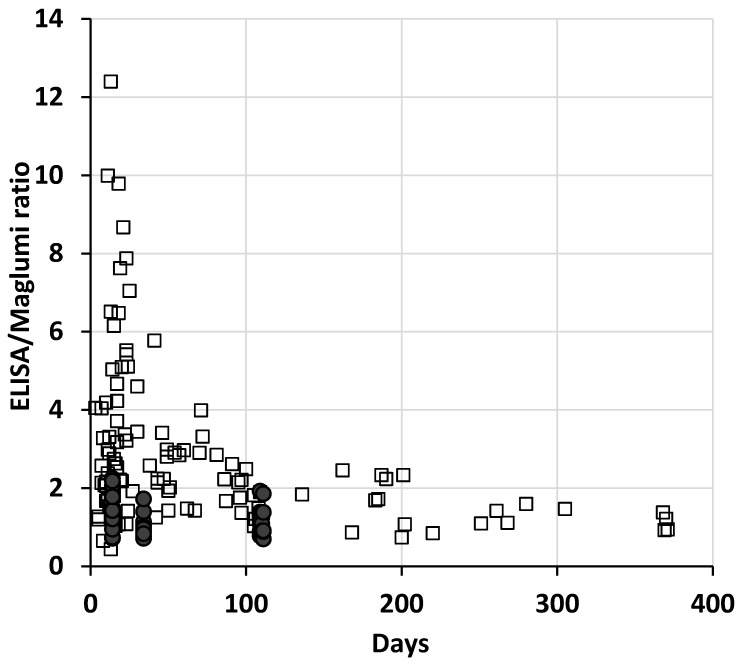
Plot of the ratios of each specimen between ELISA and Maglumi vs. the days from the onset of symptoms, or from the first inoculum of vaccine. The open squares represent the patient specimens and the filled circles the vaccinated subjects.

In the patients, the differences between methods remain elevated even after the transformation in BAU, and the dispersion of the ratios between the two methods was very high, especially in the first weeks after the onset of the disease (up to about 10–15 weeks) and reduced over time (Figure 1). Interestingly, the 13 patient specimens collected more than 200 days after symptoms show a median ratio (1.1) and a variability (10th–90th percentile: 0.86–1.57) comparable to those of vaccinated subjects after the second dose.

**Figure 2:**
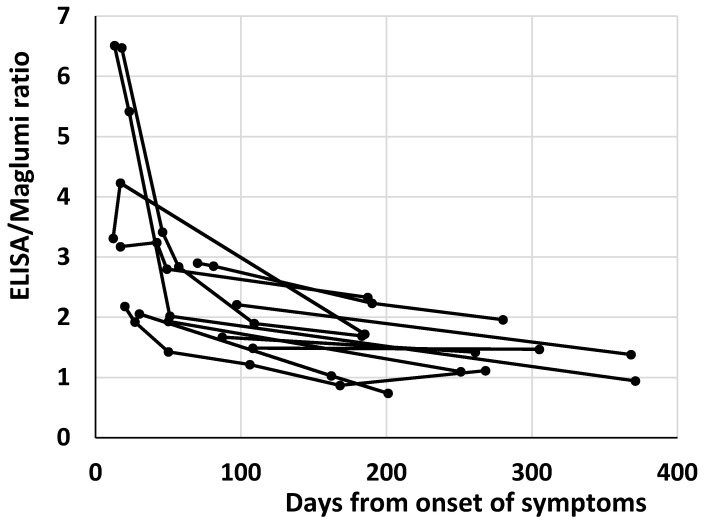
Plot of the antibody ratios between ELISA and Maglumi vs. the days from the onset of symptoms in the 12 patients with more than one sample and at least one sample over 6 months after the symptoms’ onset. Each patient is represented by a line.

The reduction of antibody heterogeneity over time probably affects the heterogeneity of antibody measurement by different methods. It is worth noting that in the 12 patients with more than one sample and at least one sample over 6 months after the symptoms’ onset, a decrease over time of the ratios between the two methods within each patient was evident (Figure 2).

A recently released Access SARS-CoV-2 IgG (1st IS) method, calibrated against the WHO standard and carried out on the DxI 800 analyzer (Beckman Coulter, Inc., Brea, CA, USA), was used to evaluate some of the cases previously studied (40 patient specimens and 31 of vaccinated subjects after the second dose), obtaining a similar behavior. 

Median ratios between DxI and Maglumi were 1.96 (10th–90th percentile: 0.5–9.1) in patients and 0.62 (10th–90th percentile: 0.46–0.87) in vaccinated subjects. Median ratios between DxI and ELISA were 0.77 (10th–90th percentile: 0.35–2.18) in patients and 0.61 (10th–90th percentile: 0.35–0.88) in vaccinated subjects. Median and percentile ratios in the 11 patients’ specimens collected after 24 weeks from the onset of symptoms were 0.83 (0.49–1.1) between DxI and Maglumi and 0.57 (0.46–1.02) between DxI and ELISA.

Despite the limited number of cases, it can be noted that the dispersion of the ratios between methods remains high in patients and narrower in vaccinated subjects. However, harmonization is not very satisfactory. Even in vaccinated subjects, there is still a difference of about 40% between DxI and the other two methods. 

In any case, the decrease in the dispersion of the ratios between methods was confirmed when patients were examined after several months from the onset of the disease.

## 3. Conclusions

The evolution of antibodies favors the idiotypes against Receptor Binding Domain, probably making the antibody population more homogeneous [7] and allowing a better harmonization between methods by using the WHO standard. 

The full realization of the harmonization seems rather complex and in certain scenarios may not be feasible.

For example, in the context of the monoclonal antibody therapies, which can be administered to patients at the onset of the disease, the possibility of dose-dependent efficacy on the basis of the levels of antibodies has been proposed [8]. However, in this case, at least according to the results described in the present study, an attempt at harmonization between methods could be unfeasible, in part due to the large variability between methods in the first months of the disease.

On the other hand, the harmonization between methods could perhaps show some efficacy in scenarios where the antibody population is more homogeneous, e.g., for monitoring the vaccinated subjects and for establishing a correlate of protection based on antibody levels [9,10]. 

## Data Availability

Data available on request from the corresponding author.
